# Supplementary material for: Water-Based Generators with Cellulose Acetate: Uncovering the Mechanisms of Power Generation
Source: Polymers (Basel). 2024 Feb 4;16(3):433. doi: 10.3390/polym16030433 (PMC10857283; doi:10.3390/polym16030433)
Supplement: Supplementary file 1 [file polymers-16-00433-s001.zip › polymers-2823829-supplementary.pdf]

polymers

MDPI

Article

Supplementary Materials

Water-Based Generators with Cellulose Acetate: Uncovering the Mechanisms of Power Generation

Seung-Hwan Lee [1,2], Hyun-Woo Lee [1], So Hyun Baek [1], Jeungjai Yun [1], Yongbum Kwon [1], Yoseb Song [1], Bum Sung Kim [1], Yong-Ho Choa [2] and Da-Woon Jeong [1,*]

[1] Korea National Institute of Rare Metals, Korea Institute of Industrial Technology, Incheon 21655, Republic of Korea; leesh93@kitech.re.kr (S.-H.L.); totptkd12@kitech.re.kr (H.-W.L.); qorthgus9@kitech.re.kr (S.H.B.); yjj0011@kitech.re.kr (J.Y.); kyb916@kitech.re.kr (Y.K.); songys88@kitech.re.kr (Y.S.); bskim15@kitech.re.kr (B.S.K.)

[2] Department of Materials Science and Chemical Engineering, Hanyang University, Ansan 15588, Republic of Korea; choa15@hanyang.ac.kr

* Correspondence: dwjeong@kitech.re.kr; Tel.: +82-32-226-1362; Fax: +82-32-226-1374

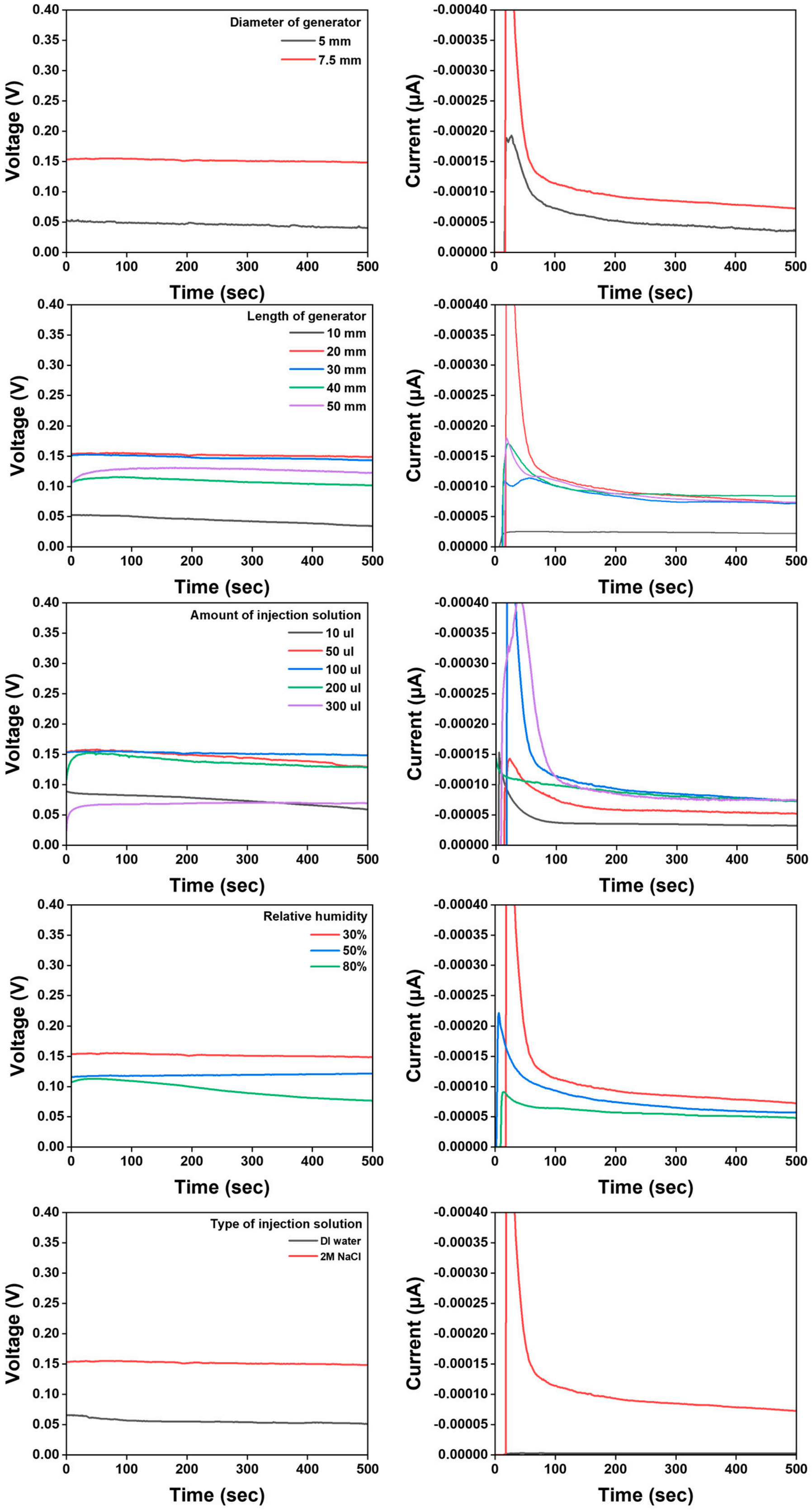

Figure S1. Raw data for organizing power generation performance data in the manuscript.

Photal

ELSZ
Common

Intensity Distribution S/N :

User	: Common	Group	: 이승환	Repetition : 1/1
Date	: 2023-12-06	File Name	: C.B	
Time	: 15:15:07	Sample Information	:	
SOP Name	: Sizing(dispo-low)			Security : No Security

Version 3.600 / 2.30

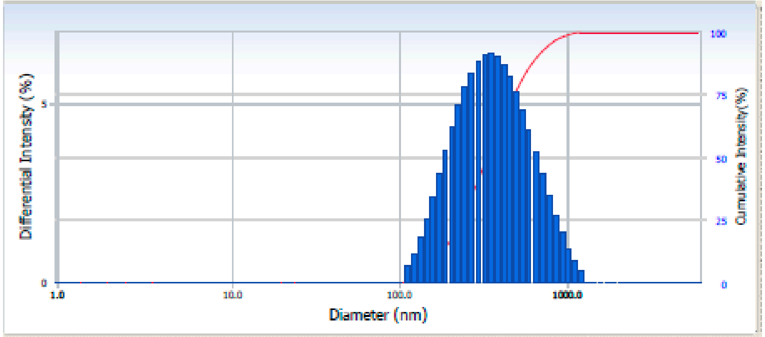

Distribution Results (Contin)

Peak	Diameter (nm)	Std. Dev.
1	416.2	215.1
2	0.0	0.0
3	0.0	0.0
4	0.0	0.0
5	0.0	0.0
Average	416.2	215.1

Residual : 1.330e-002 (O.K)

Cumulants Results

Diameter	(d)	: 341.5	(nm)
Polydispersity Index	(P.I.)	: 0.239	
Diffusion Const.	(D)	: 1.823e-008	(cm^2/sec)

Measurement Condition

Temperature	: 25.0	(°C)
Diluent Name	: WATER	
Refractive Index	: 1.3272	
Viscosity	: 0.7015	(cP)
Scattering Intensity	: 14485	(cps)
Attenuator 1	: 11.3	(%)

Intensity Distribution Table

d (nm)	f(%)	f(cum.%)	d (nm)	f(%)	f(cum.%)	d (nm)	f(%)	f(cum.%)	d (nm)	f(%)	f(cum.%)
1.0	0.0	0.0	9.0	0.0	0.0	81.6	0.0	0.0	737.4	3.0	92.3
1.1	0.0	0.0	9.9	0.0	0.0	89.1	0.0	0.0	805.2	2.5	94.8
1.2	0.0	0.0	10.8	0.0	0.0	97.3	0.0	0.0	879.3	1.9	96.7
1.3	0.0	0.0	11.8	0.0	0.0	106.3	0.0	0.0	960.2	1.4	98.1
1.4	0.0	0.0	12.8	0.0	0.0	116.1	0.5	0.5	1048.6	1.0	99.0
1.6	0.0	0.0	14.0	0.0	0.0	126.8	0.8	1.3	1145.1	0.6	99.7
1.7	0.0	0.0	15.3	0.0	0.0	138.4	1.3	2.5	1250.5	0.3	100.0
1.9	0.0	0.0	16.7	0.0	0.0	151.2	1.8	4.4	1365.6	0.0	100.0
2.0	0.0	0.0	18.3	0.0	0.0	165.1	2.4	6.8	1491.3	0.0	100.0
2.2	0.0	0.0	20.0	0.0	0.0	180.3	3.1	9.8	1628.5	0.0	100.0
2.4	0.0	0.0	21.8	0.0	0.0	196.9	3.7	13.6	1778.4	0.0	100.0
2.6	0.0	0.0	23.8	0.0	0.0	215.0	4.4	17.9	1942.1	0.0	100.0
2.9	0.0	0.0	26.0	0.0	0.0	234.8	4.9	22.8	2120.8	0.0	100.0
3.1	0.0	0.0	28.4	0.0	0.0	256.4	5.5	28.3	2316.0	0.0	100.0
3.4	0.0	0.0	31.0	0.0	0.0	280.0	5.9	34.2	2529.1	0.0	100.0
3.7	0.0	0.0	33.8	0.0	0.0	305.7	6.2	40.3	2761.9	0.0	100.0
4.1	0.0	0.0	37.0	0.0	0.0	333.9	6.3	46.7	3016.1	0.0	100.0
4.5	0.0	0.0	40.4	0.0	0.0	364.6	6.4	53.1	3293.7	0.0	100.0
4.9	0.0	0.0	44.1	0.0	0.0	398.1	6.3	59.4	3596.8	0.0	100.0
5.3	0.0	0.0	48.1	0.0	0.0	434.8	6.1	65.5	3927.8	0.0	100.0
5.8	0.0	0.0	52.6	0.0	0.0	474.8	5.8	71.2	4289.3	0.0	100.0
6.4	0.0	0.0	57.4	0.0	0.0	518.5	5.3	76.6	4684.1	0.0	100.0
6.9	0.0	0.0	62.7	0.0	0.0	566.2	4.8	81.4	5115.2	0.0	100.0
7.6	0.0	0.0	68.4	0.0	0.0	618.3	4.3	85.6	5585.9	0.0	100.0
8.3	0.0	0.0	74.7	0.0	0.0	675.2	3.7	89.3	6100.0	0.0	100.0
D (10%) :	181	(nm)	D (50%) :	349.4	(nm)	D (90%) :	689.4	(nm)			

Figure S2. Intensity distribution results of coating solution (original data).
